# The relevance of polycomb group proteins to the development of psychiatric disorders

**DOI:** 10.3389/fcell.2022.927833

**Published:** 2022-07-22

**Authors:** Jacob Peedicayil

**Affiliations:** Department of Pharmacology and Clinical Pharmacology, Christian Medical College, Vellore, India

**Keywords:** polycomb, psychiatric disorder, pathogenesis, psychiatry, development

## Introduction

Proteins belonging to the Polycomb Group (PcG) are a widely-recognized category of proteins that modify chromatin. PcG proteins and the triothorax group (TrxG) of proteins dynamically and antagonistically regulate gene expression with PcG proteins inhibiting, and the TrxG proteins stimulating, gene expression (Kuehner and Yao, 2019). PcG proteins are usually classified into a pair of distinct multiprotein groups, polycomb repressive complex 1 (PRC1) and PRC2 (Blackledge and Klose, 2021; Piunti and Shilatiford, 2021). The two classes have different enzymatic properties, although both are usually associated with silencing of transcription. PRC1 has E3 ubiquitin ligase action and in mammalian cells specifically catalyzes Lys118 and Lys119 of histone 2A, and mainly causes monoubiquitylation of them (Piunti and Shilatiford, 2021). PRC1 is not one complex, but comprises a minimum of eight varying complexes having different assortments of subunits. These subunits give differing biological functions to the complexes. The various complexes may be additionally classified as canonical or non-canonical members of PRC1, depending on their possessing a CBX protein or either of a pair of homologous proteins, namely RING1 and YY1 binding protein called RYBP, and YY1-associated factor 1 (YAF2), respectively. Non-canonical and canonical PRC1 complexes possess the molecule really interesting new gene 1A (RING1A), a protein, or its homologue, RING1B, that gives PRC1 its catalytic property (Piunti and Shilatiford, 2021).

The PRC2 complex comprises a methyltransferase taking part in monomethylating, dimethylating, and trimethylating histone H3 Lys27. The enzymatic properties of PRC2 depend on the following subunits of the complex: 1) embryonic ectoderm development (EED), 2) suppressor of zeste 12 (SUZ12), and 3) enhancer of zeste homologue 2 (EZH2), or its homologue EXH1. In addition to RB binding protein 4 (RBBP4) or RBBP7, these subunits comprise the subunits of PRC2 that comprise its core, and along with several other subunits make up the recently elucidated complex variations of PRC2 (Piunti and Shilatiford, 2021).

Transcriptional repression occurs due to the enzymatic functions of PRC1 and PRC2, particularly the generation of H2AK119ub and H3K27me3, respectively. However, both PRC1 and PRC2 have been shown to also have non-catalytic functions that facilitate repression of transcription (Piunti and Shilatiford, 2021). There is a sophisticated pathway that regulates the bringing of such proteins to regulatory regions of the genome called PcG and trxG response elements (PRE and TRE). After being brought to their targets, multimeric PcG and trxG protein complexes regulate gene transcription by modulation of the structure of chromatin, especially by the placement of specific histone modifications, control of chromatin accessibility, and controlling the 3-D nuclear composition of PRE and TRE (Grimaud et al., 2006).

## The relevance of polycomb group proteins to the development of psychiatric disorders

During brain development, proteins belonging to the PcG and trxG groups make multimeric complexes playing important roles in controlling gene expression in neurons and regulating epigenetic changes (Kuehner and Yao, 2019). PcG proteins are thought to play key roles in the regulation of the growth and differentiation of neural stem and progenitor cells (Bölicke and Albert, 2022). While studying the relevance of epigenetics to the pathogenesis of psychiatric disorders, considerable attention has been given to epigenetic mediators like DNA methylation and histone modifications (Lee and Avramopoulos, 2021), while the relevance of PcG proteins has been neglected. Here, I give examples of how malfunctioning of PcG proteins can predispose to psychiatric disorders.

## The relevance of polycomb group proteins to the development of organic psychiatric disorders

PcG proteins are thought to take part in the development of organic psychiatric disorders (Table 1). Weaver syndrome is an uncommon congenital disorder recorded in two families in 1974 (Gibson et al., 2012). Intellectual disability (ID) is widely prevalent in patients with this condition. *De novo* mutations in EZH2 are the cause. EZH2 is a histone methyltransferase that acts as the enzymatic molecule of PRC2 to cause inhibition of gene expression due to methylation of lysine 27 on histone H3, that is, H3K27 (Cohen at al. 2016).

**TABLE 1:** Types of psychiatric disorders.

Organic psychiatric disorders	Non-organic (functional) psychiatric disorders
(Psychiatric disorders due to transient or permanent brain dysfunction caused by underlying lesion in the brain)	(Psychiatric disorders not due to any factors such as aging, trauma, and tumours)
Intellectual disability like fragile X mental retardation	Anxiety disorder
Autism spectrum disorders	Major depressive disorder
Alzheimer’s dementia	?Schizophrenia

Using a cell line from a patient with fragile X syndrome, Kumari and Usdin (2014) demonstrated that trimethylation levels of histone H3 on lysine 27 of the FMR1 gene, which reflects EZH2 activity, is increased after the activation of the allele silenced by the DNA hypomethylating agent azacitidine or the SIRT1 inhibitor splitomicin. They found that the level of H3K27me3 increases or decreases in parallel with the FMR1 mRNA level. Moreover, decreasing the levels of FMR1 mRNA decreased the accumulation of H3K27me3. Hence, this represents a model for FMR1 gene silencing in which the FMR1 mRNA produced from the reactivated allele acts *in cis* to supress its own expression by bringing PcG complexes to the FMR1 locus.

Von Schimmelmann et al. (2016) showed that PRC2, which favours neuron specification during differentiation, helps in the suppression of a transcriptional programme that adversely affects adult neuron function and survival. They showed that PRC2 deficiency in striatal neurons leads to de-repression of selected, mainly bivalent, PRC2 target genes which are dominated by self-regulating transcription factors normally suppressed in such neurons. The transcriptional changes in PRC2-deficient neurons led to increasing and fatal neurodegeneration in mice. Hence, this data indicates an important role for PRC2 in protecting neurons from degeneration. The same group (Ayata et al., 2018) later showed that microglia clearance activity of dying neurons and non-functional synapses in the adult brain is regionally regulated and depends on the rate of neuronal attrition. Cerebellar microglia, but not striatal or cortical microglia, demonstrated increased levels of basal clearance activity, which correlated with greater cerebellar neuronal attrition. Exposing forebrain microglia to apoptotic cells stimulated gene expression programmes augmenting clearance activity. The authors demonstrated that PRC2 curtails expression of genes that augment clearance activity in striatal and cortical microglia by epigenetic mechanisms. Loss of PRC2 caused abnormal stimulation of the microglia clearance phenotype, leading to changes in structure and activity of neurons. Hence, this work demonstrates an important role for epigenetics in reducing microglia-induced neuronal changes which are commonly linked to organic psychiatric disorders.

The PcG protein BMi1 controls compaction of chromatin and silencing of genes. The expression of BMi1 is adequate in neurons of adult brains but is decreased in the brains of patients with Alzheimer’s disease (AD). El Hajjar et al. (2019) found that mice without one allele of Bmi1 (Bmi1/-) normally develop but later have age-related cognitive deficits and neurodegeneration, sharing similarities with AD. Bmi1^+/−^mice also transgenic for the amyloid *β* precursor protein had severe disease and died prematurely. Heterochromatin loss and DNA damage response (DDR) at repetitive DNA sequences were very common in Bmi1^+/−^mouse neurons and inhibition of the DDR lessened the amyloid and tau phenotype. Heterochromatin abnormalities and DDR at repetitive DNA sequences were also observed in brains of AD patients. Thus, the authors suggest that aging Bmi1^+/−^mice may be a suitable model to elucidate and investigate new mechanisms in relation to the development of AD.

The gene encoding activator of transcription and developmental regulator (AUTS2) is linked to many organic psychiatric disorders including autism spectrum disorders and ID. Monderer-Rothkoff et al. (2021) showed in mouse neuro2a cells that when neuronal differentiation is initiated, there is a shift in expression from a long AUTS2 isoform to a short AUTS2 isoform. A yeast two-hybrid screen identified the splicing factor SF3B1 as an interactor of both isoforms. However, the PcG proteins, PCGF3, and PCGF5, were found to interact only with the long AUTS2 isoform. Reporter assays showed that the first exons of the long AUTS2 isoform function as a transcription repressor, but the part comprising the short isoform acts as a transcriptional repressor. The expression levels of PCGF3 influenced the ability of the long AUTS2 isoform to activate or repress transcription. Mouse embryonic stem cells (mESCs) with heterozygote mutations in Auts2 had increased cell death during *in vitro* corticogenesis, and this was corrected by overexpressing the human AUTS2 transcripts. mESCs with a truncated AUTS2 protein demonstrated premature neuronal differentiation, whereas cells overexpressing AUTS2, especially the long transcript, demonstrated increased expression of markers of pluripotency and differentiation that was delayed.

PcG protein activity and DNA methylation, both of which inhibit gene expression, influence each other. For example, in vertebrates, EZH2 interacts with three DNA methyltransferases (DNMTs), and is needed both for the binding of the DNMTs, and for CpG methylation, at EZH2-targeted promoters (Bantignies et al., 2006). This data is supported by the finding of Zhang et al. (2020) who investigated DNA methylation across the genome in post-mortem prefrontal cortex samples of AD patients, and found that DNA hypermethylation in the prefrontal cortex of AD patients was significantly over-represented in PcG-repressed regions.

## The relevance of polycomb group proteins to the development of non-organic psychiatric disorders

PcG proteins are also incriminated in the development of non-organic (functional) psychiatric disorders (Table 1). Early life stress (ELS) is a well known factor that can lead to psychiatric disorders in adults, especially non-organic psychiatric disorders (Carr et al., 2013). Murgatroyd and Spengler (2014) employed a model of hypothalamic-like differentiation derived from embryonic stem cells along with *in vivo* experiments to demonstrate that the binding of PcG protein complexes occurred before the development of changes in methylation of DNA due to ELS that correlated with gene silencing. At the same time, PcG occupancy correlated with the TeT enzyme presence so that DNA methylation did not occur. Initial hypothalamic-like differentiation caused PcG removal, DNMT recruitment and enhancer methylation. Simultaneously, binding of methyl-CpG-binding protein increased at the enhancer even though expression of arginine vasopressin (Avp) during the later stages of differentiation and the perinatal period continued to rise. Hence, the authors suggest that there is a new role for PcG proteins in stimulating DNA methylation in response to ELS at the Avp enhancer before epigenetic programming, and that this suggests that PcG proteins belong to a changeable silencing system during development of neurons.

In genome-wide association studies (GWAS) in schizophrenia (SZ), top findings demonstrate indirect support for a role of PcG proteins in SZ pathogenesis: MIR137 is a SZ genome-wide risk gene. An experimentally validated target of MIR137 is EZH2. Szulwach et al. (2010) showed in adult neural stem cells that miR-137 post-transcriptionally represses expression of EZH2. Another locus associated with SZ is the CACNA1C locus which codes for Cav1.2, 1 of 4 subunits of the L-type voltage-gated calcium channel. There are changes in non-coding regions of this locus which interact with the promoter and are proven expression quantitative trait loci. Using reporter gene constructs Billingsley et al. (2018) showed that the promoter of the CACNA1C locus is a major mediator of inducible regulation of this locus in the SH-SY5Y neuroblastoma cell line. Initial interrogation of ENCODE Chip-seq data over the CACNA1C promoter suggested binding of EZH2, which agreed with the authors’ data that overexpression of EZH2 represses this gene. Array data obtained from the Human Brain Transcriptome showed that EZH2 is markedly present throughout the developing brain, but later maintained at low levels after birth and adulthood. RNA-seq data has shown a 3-fold increase in the expression of EZH2 within the anterior cingulate cortex of SZ patients. Hence the authors suggest that EZH2 may lead to the development of SZ at two different time points - by development disruption causing changes in neurodevelopment, or by abnormal reactivation of gene expression within the adult brain.

Cohen et al. (2017) investigated a novel molecular mechanism shaping anxiety-like behaviour in rats bred specifically for alterations in emotionality and stress reactivity. The authors found that the microRNA, miR-101a-3p and its target Ezh2 in the amygdala, are involved in rat anxiety-like behaviour. High novelty responding (HR) rats displayed low anxiety and had low levels of miR-101a-3p in the amygdala in comparison with low novelty responding (LR) rats that usually display high trait anxiety. In order to find out if there is a causal relationship between amygdalar miR-101a-3p and anxiety-like behaviour, the authors used a viral approach to overexpress miR-101a-3p in the amygdala of HR rats and test whether it would increase their usual low levels of behaviour suggestive of anxiety. It was observed that raising miR-101a-3p in the amygdala increases HR’s features resembling anxiety in the open-field test and elevated plus maze. Viral-mediated miR-101a-3p overexpression also decreased expression of Ezh2. Ezh2 knockdown with short-interfering RNA also increased HR’s anxiety-like behaviour, but to a lower level than with miR-101a-3p overexpression. Hence, raising miR-101a-3p expression in the amygdala raises anxiety-like behaviour and this is at least partly caused by the repression of Ezh2.

In conclusion, there is accumulating evidence that PcG proteins play a major role in the pathogenesis of organic and non-organic psychiatric disorders. The study of the role of PcG proteins in psychiatric research has been relatively neglected, and should be given due attention henceforth.
